# Randomized Trial—PrEscription of intraDialytic exercise to improve quAlity of Life in Patients Receiving Hemodialysis

**DOI:** 10.1016/j.ekir.2021.05.034

**Published:** 2021-05-30

**Authors:** Sharlene A. Greenwood, Pelagia Koufaki, Jamie H. Macdonald, Sunil Bhandari, James O. Burton, Indranil Dasgupta, Kenneth Farrington, Ian Ford, Philip A. Kalra, Sharon Kean, Mick Kumwenda, Iain C. Macdougall, Claudia-Martina Messow, Sandip Mitra, Chante Reid, Alice C. Smith, Maarten W. Taal, Peter C. Thomson, David C. Wheeler, Claire White, Magdi Yaqoob, Thomas H. Mercer

**Affiliations:** 1 Renal Medicine, King’s College Hospital NHS Trust, London, UK; 2 School of Renal Medicine, King’s College London, London, UK; 3 School of Health Sciences, Queen Margaret University, Edinburgh, UK; 4 School of Sport, Health and Exercise Sciences, Bangor University, Wales, UK; 5 Renal Medicine, Hull University Teaching Hospitals NHS Trust, Hull, UK; 6 Department of Cardiovascular Sciences, University of Leicester, Leicester, UK; 7 Renal Medicine, University Hospital Birmingham NHS Foundation Trust, Birmingham, UK; 8 Renal Medicine, Lister Hospital, Stevenage, UK; 9 Robertson Centre for Biostatistics, University of Glasgow, Glasgow, UK; 10 Renal Medicine, Salford Royal Hospital, Salford, UK; 11 Renal Medicine, Glan Clwyd Hospital, Wales, UK; 12 Renal Medicine, Manchester University Hospitals, Manchester, UK; 13 Department of Health Sciences, University of Leicester, Leicester, UK; 14 Division of Medical Sciences and Graduate Entry Medicine, University of Nottingham, Nottingham, UK; 15 Renal Medicine, Queen Elizabeth University Hospital, Glasgow, UK; 16 Renal Medicine, University College London, London, UK; 17 The George Institute for Global Health, New South Wales, Australia; 18 Renal Medicine, The Royal London Hospital, London, UK

**Keywords:** chronic kidney disease, physical activity, physical function, rehabilitation

## Abstract

**Introduction:**

Whether clinically implementable exercise interventions in people receiving hemodialysis (HD) therapy improve health-related quality of life (HRQoL) remains unknown. The PrEscription of intraDialytic exercise to improve quAlity of Life **(**PEDAL) study evaluated the clinical benefit and cost-effectiveness of a 6-month intradialytic exercise program.

**Methods:**

In a multicenter, single-blinded, randomized, controlled trial, people receiving HD were randomly assigned to (i) intradialytic exercise training (exercise intervention group [EX]) and (ii) usual care (control group [CON]). Primary outcome was change in Kidney Disease Quality of Life Short-Form Physical Component Summary (KDQOL-SF 1.3 PCS) from baseline to 6 months. Cost-effectiveness was determined using health economic analysis; physiological impairment was evaluated by peak oxygen uptake; and harms were recorded.

**Results:**

We randomized 379 participants; 335 and 243 patients (EX *n* = 127; CON *n* = 116) completed baseline and 6-month assessments, respectively. Mean difference in change PCS from baseline to 6 months between EX and CON was 2.4 (95% confidence interval [CI]: −0.1 to 4.8) arbitrary units (*P* = 0.055); no improvements were observed in peak oxygen uptake or secondary outcome measures. Participants in the intervention group had poor compliance (47%) and poor adherence (18%) to the exercise prescription. Cost of delivering intervention ranged from US$598 to US$1092 per participant per year. The number of participants with harms was similar between EX (*n* = 69) and CON (*n* = 56). A primary limitation was the lack of an attention CON. Many patients also withdrew from the study or were too unwell to complete all physiological outcome assessments.

**Conclusions:**

A 6-month intradialytic aerobic exercise program was not clinically beneficial in improving HRQoL as delivered to this cohort of deconditioned patients on HD.

## Graphical abstract

**Figure:**
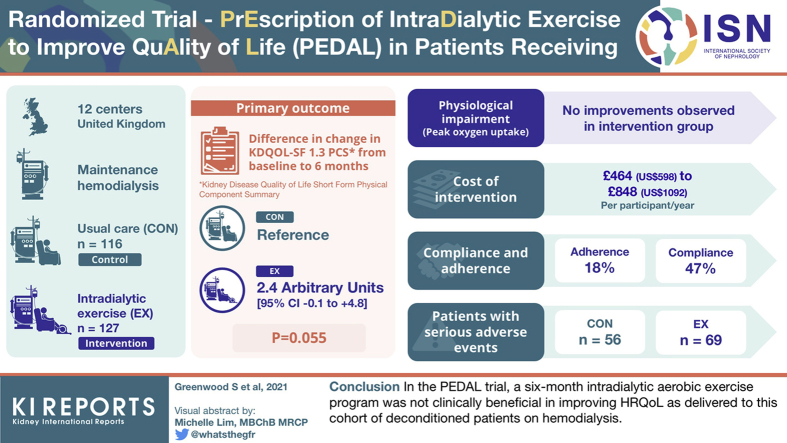

## Introduction

Improved HD techniques and management of coexisting disease has improved the average life expectancy of patients receiving HD therapy globally, but disability and associated symptoms remain highly prevalent accounting for more life years lost to disability.^1^ In the UK, 48% of the HD population report severe functional dependencies,^2^ which impact on HRQoL.^3^ Components of HRQoL, particularly the domain of physical functioning, stand out as the strongest predictor of survival, hospitalizations, and morbidity.^4^ Knight *et al.*^5^ and Lowrie *et al.*^6^ report multiple symptoms that affect the physical component of HRQoL.^7^ Moreover, higher levels of physical activity are associated with better scores in HRQoL measures, physical functioning, depression, and burden of kidney disease symptoms.^8^

The physical component of HRQoL, therefore, may be targeted with interventions to enhance physical activity. In patients receiving HD therapy, systematic reviews indicate that a range of exercise training interventions improve physical function and alleviate disability symptoms.^10^, ^11^, ^12^, ^13^, ^14^, ^15^, ^16^, ^17^, ^18^, ^19^, ^20^, ^21^, ^22^, ^9^ Of particular interest are studies investigating intradialytic exercise, as the environment of unit-based HD provides a platform for longer-term sustainable implementation of exercise rehabilitation programs.^23^ The pre-existing need for patients to attend for standard thrice weekly, 4 hour-long HD sessions provides an opportunity to deliver a structured and supervised rehabilitation program with reduced patient burden regarding time, effort, and travel costs.^24^^,^^25^ Thus, physical activity behaviors could be promoted using an implementation model that integrates physical activity into the main health care system for patients receiving HD therapy.

Nevertheless, very few dialysis units have chosen to implement this physical rehabilitation option in the UK. A barrier to implementation has been a lack of high-quality, adequately powered randomized controlled trials of intradialytic exercise with patient-reported outcomes (HRQoL), health economics (cost-effectiveness), and harms (serious adverse events [SAEs]) as the primary outcomes. Thus, the balance of benefits to costs and harms has been impossible to evaluate. Consequently, the PrEscription of intraDialytic exercise to improve quAlity of Life **(**PEDAL) trial was commissioned by the National Institute for Health Research to evaluate whether intradialytic exercise was able to improve HRQoL in patients receiving HD therapy. The primary objective was to determine, in stage 5 chronic kidney disease (CKD) patients receiving maintenance HD, whether usual care augmented by intradialytic exercise training for a period of 6 months improved KDQOL-SF 1.3 PCS.

## Methods

### Trial Design and Oversight

We conducted this pragmatic prospective randomized controlled trial in 5 regions (London, Scotland, Wales, North-West England, and Midlands), across a total of 12 HD units, in the UK. The trial recruited prevalent patients with stage 5 dimensions of CKD receiving HD therapy. Briefly, the intervention consisted of using a modified cycle ergometer to perform aerobic exercise in a semirecumbent position, 3 times per week during the first 2 hours of HD. Twice per week, after the aerobic cycling exercise, participants completed lower extremity muscular conditioning exercises. These included 3 sets of 10 to 15 repetitions of dynamic resistance exercises for all major muscle groups. All exercises were performed against body weight before progression with ankle weights and TheraBands (Akron, OH). The exercise program was delivered and supervised by physiotherapy assistants.

London Fulham Research Ethics Committee approved the protocol (14/LO/1851), and all the participants provided written informed consent. The study was registered prospectively (ISRCTN N83508514). The trial protocol and details on inclusion/exclusion criteria, randomization procedure, and exercise intervention and prescription have been described elsewhere.^26^ The Consolidated Standards of Reporting Trials Extension for Patient Report Outcomes also suggested reporting all the multi-item scales from the KDQOL-SF instrument.

### Primary Outcome

The primary outcome for this study was the change in KDQOL-SF 1.3 PCS from baseline to 6 months.^27^ The KDQOL-SF 1.3 instrument was chosen because of its validity in patients with CKD and inclusion of a generic core that has been widely used in CKD and other populations. The KDQOL-SF 1.3 is a disease-specific QoL measure that includes 43 kidney disease–targeted items and 36 items providing a generic core and an overall health-rating item. The questionnaire was completed by patients using pen and paper, with queries answered by research officers blinded to treatment allocation. Scoring followed currently recommended methods.^28^ Thus, the PCS score can be interpreted as follows: a score above or below 50 is above or below the average, respectively, in the US general population, whereas a 1-point difference in the score is one-tenth of a SD. Analysis of within-trial change in the KDQOL-SF 1.3 PCS score from baseline, adjusted for baseline levels and randomization minimization variables, suggested that the study had 80% power to detect a 4-point difference with only 87 participants per group (with complete data at baseline and 6-month follow-up).

### Secondary Outcomes

#### HRQoL, Cost-Effectiveness, and Harms

From the KDQOL-SF 1.3, the multi-item scale of energy/fatigue and the kidney disease–targeted items (burden of kidney disease) were presented as prespecified. In addition, the remaining 7 multi-item scales were presented. Then, a generic preference-based measure of HRQoL was obtained using the EuroQol 5-dimension descriptive system (EQ-5D-5L).^29^ The EQ-5D-5L comprises the following 5 dimensions: mobility, self-care, usual activities, pain/discomfort, and anxiety/depression. The EQ—visual analog scale was also obtained, whereby participants reported their self-rated evaluation of their health state on a 0 to 100 visual analog scale. Costs of delivering the PEDAL intervention were calculated, including exercise equipment, assumed to cost £1000 with a lifetime of 10 years and maintenance costs of £50 per year. Staff costs were assumed to include one ×0.6 full-time equivalent physiotherapy assistant (mid band 4 Agenda for Change scale, annual employer costs from £25,866 outside London to £34,787 in London) per 12 to 20 participants (to reflect different geographic spacing of kidney units in rural and urban areas) and one ×1.0 full-time equivalent supervisor (mid band 8 Agenda for Change, annual employer costs from £55,078.00 outside London to £71,418.96 in London) per 80 participants.

#### Physical Function

Upper limits of exercise tolerance were assessed by peak oxygen uptake determined by an incremental cycling protocol.^26^ Physical function limitations were assessed by the sit-to-stand-60^30^ and gait speed in 10 m.^31^ Physical activity behaviors were captured by the International Physical Activity Questionnaire Short-Form^32^; ability to undertake activities of daily livings was recorded by the Duke Activity Status Index^33^; and fear of falling was assessed by the Tinetti Falls Efficacy Scale.^34^

#### Cardiovascular Risk and Clinical Measures

Arterial stiffness was assessed by the carotid–femoral pulse wave velocity,^35^ measured using the Vicorder system (Skidmore Industries, UK) and by following the current recommendations.^35^ Measures of body mass index and waist circumference were also recorded. Clinical data included cause of kidney disease, comorbidities, routine clinical blood tests (hemoglobin, serum phosphate, and parathyroid hormone), and medications (including erythropoiesis-stimulating agents).

#### Harms

Harms were actively recorded in both groups by the physiotherapy assistants from baseline to the end of the 6-month follow-up period (*n* = 335). Relationship to the intervention was evaluated by the lead clinician at each center, who was not blinded to treatment allocation. SAEs were reviewed by a data safety monitoring committee; rules for stopping the trial were that the committee identified a marked increase in expected or unexpected SAEs owing to the testing or intervention procedures. Data on hospitalizations and deaths (all-cause mortality and cardiovascular mortality) were collected by reviews of clinical databases and records at each study visit.

#### Compliance and Adherence (Fidelity) to Exercise Prescription

General compliance was recorded as the percentage of exercise sessions completed out of the total prescribed for the 6-month follow-up period. Adherence (fidelity) was recorded as the percentage of patients who adhered exactly to the prescribed exercise (cycling and muscle conditioning exercises) at the prescribed intensity and cycling time duration for each session across the 6 months. In addition, the percentage of patients who temporarily (>2 weeks) paused exercise was noted. These data were recorded by physiotherapy assistants through completion of sessional exercise diaries.

### Statistical Analyses

The primary outcome measure (change from baseline to 6 months in KDQOL-SF 1.3 PCS) was compared between the control and intervention groups using a normal linear model adjusting for baseline KDQOL-SF 1.3 PCS and the randomization minimization variables (age, gender, diabetes status). The findings are presented as the adjusted mean difference (95% CI) between the treatment groups. Significance was set at *P* ≤ 0.05. The main analysis was carried out on research participants with PCS assessments at baseline and 6 months. A total of 2 sensitivity analyses were also carried out, first imputing a score of 0 for those who died before 6 months and second based on all participants with a baseline PCS using the method of multiple imputation. As results were consistent between methods, only the main analysis is reported herein.

Secondary continuous outcomes were analyzed as for the primary outcome. For health economic data, we estimated the mean between-group difference in costs of the intervention and the mean between-group difference in quality-adjusted life years accrued by participants during the study, estimated as the area under the health utility curve from study entry (i.e., randomized and attended baseline visit) to follow-up (6 months after). Costs in the CON were set to 0. Estimated between-group differences in cost and quality-adjusted life years were obtained by the method of recycled prediction in 5000 bootstrap samples. The distribution of these quantities was summarized and presented graphically in the incremental cost effectiveness plane. Time-to-event outcomes (cardiovascular and all-cause mortality) were calculated as time from randomization and were compared between treatment groups using Cox proportional hazard regression models. The results are reported as the adjusted hazard ratio for intervention versus control (95% CI). Data involving counts of events (hospitalizations) were compared between treatment groups using negative binomial regression models adjusting for length of follow-up. The results are reported as adjusted rate ratio (95% CI). Harms (SAEs) were tabulated by system organ class and body system using the Medical Dictionary for Regulatory Activities Terminology.^36^ Recurrent events were counted separately. Compliance and adherence data were tabulated and presented visually.

## Results

**Figure 1:**
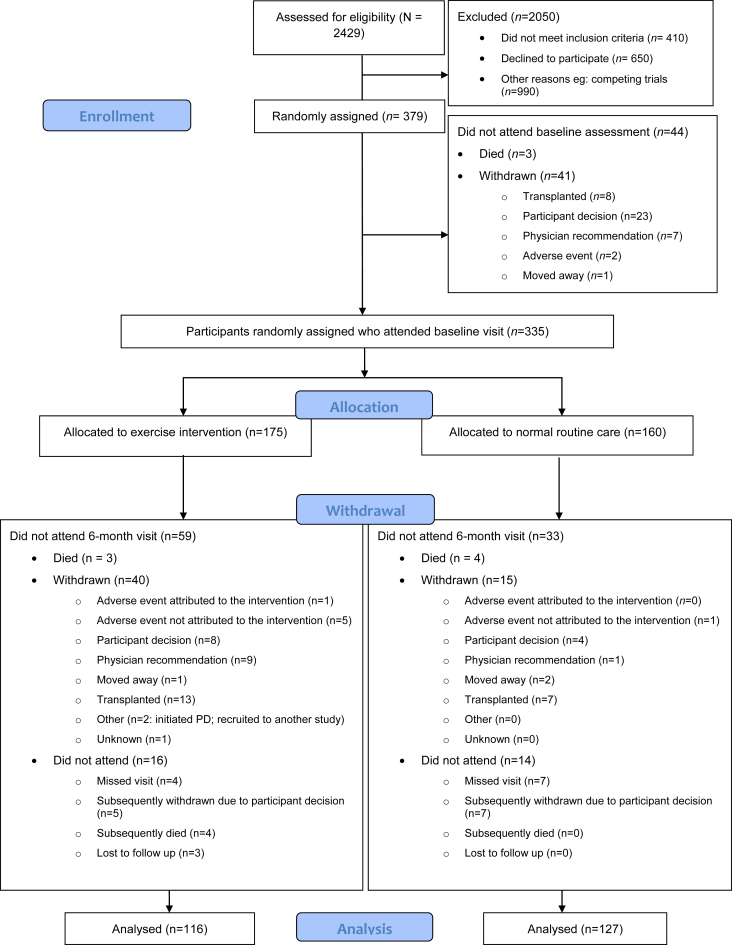
CONSORT diagram of the flow of patients across the various phases of the trial. CONSORT, Consolidated Standards of Reporting Trials; PD, progression disease.

Patient flow, including recruitment to and retention in the trial, is detailed in Figure 1. A total of 2409 patients were screened for eligibility. Nevertheless, 410 were not eligible per inclusion criteria, 660 patients declined to participate, and 990 patients were not eligible to participate owing to competing trials in this same population within the UK. A total of 335 participants attended a baseline study visit, 175 patients who were randomized to EX and 160 participants to CON. The primary outcome was known for 243 participants (73%) who attended a baseline visit, 116 participants (66%) in the exercise group, and 127 participants (79%) in the usual care group. More patients withdrew from EX (40; 34.5%) than CON (15; 11.8%) owing to participant decision, physician recommendation because of medical concerns, and transplantation. Apart from an increased number of smokers in the group of patients who withdrew from EX, no obvious differences in characteristics of the withdrawn and not withdrawn groups were present (Table 1).

**Table 1:** Baseline characteristics of all patients in the trial, stratified by group, and according to withdrawal from the trial

Baseline characteristic	CON, not withdrawn		EX, not withdrawn		CON, withdrawn		EX, withdrawn	
	*N*	Summary	*N*	Summary	*N*	Summary	*N*	Summary
Age								
Mean (SD)	145	59.8 (14.1)	135	60.5 (15.0)	15	52.8 (19.9)	40	56.8 (13.3)
Median (Q1, Q3)		59.7 (50.5, 71.0)		62.1 (47.9, 72.9)		56.1 (34.7, 61.2)		56.3 (49.6, 64.3)
Gender								
*n* (%) Female	145	55 (38)	135	56 (42)	15	4 (27)	40	11 (28)
Ethnicity								
*n* (%) White	145	67 (46)	135	73 (54)	15	10 (67)	40	19 (48)
*n* (%) Black Caribbean		26 (18)		17 (13)		1 (7)		3 (8)
*n* (%) Black African		33 (23)		24 (18)		1 (7)		10 (25)
*n* (%) South Asian		15 (10)		16 (129)		2 (13)		6 (15)
*n* (%) Chinese		1 (1)		1 (1)		0 (0)		0 (0)
*n* (%) Other^a^		3 (2)		4 (3)		1 (7)		2 (5)
Weight (kg)								
Mean (SD)	143	80.8 (20.5)	135	79.2 (18.8)	15	82.5 (13.8)	40	82.8 (24.8)
Median (Q1, Q3)		77.0 (66.1, 92.2)		76.4 (65.4, 90.8)		83.0 (67.5, 91.5)		78.5 (67.4, 90.7)
BMI (kg/m^2^)								
Mean (SD)	143	28.8 (6.5)	135	28.5 (6.5)	15	28.8 (5.5)	40	29.2 (8.8)
Median (Q1, Q3)		28.0 (24.5, 32.0)		27.0 (23.8, 32.2)		27.8 (24.2, 32.4)		27.6 (22.3, 32.6)
Smoking								
n (%) Current	145	19 (13.1)	135	18 (13.3)	15	0 (0.0)	40	5 (12.5)
n (%) Former		45 (31.0)		39 (28.9)		4 (26.7)		10 (25.0)
n (%) Never		81 (55.9)		78 (57.8)		11 (73.3)		25 (62.5)
SBP (mm Hg)								
Mean (SD)	142	138.6 (23.4)	135	134.4 (21.3)	15	133.9 (22.6)	40	134.1 (17.5)
Median (Q1, Q3)		138.0 (121.8, 153.9)		133.7 (121.3, 147.5)		130.0 (115.0, 152.2)		131.5 (121.0, 142.8)
DBP (mm Hg)								
Mean (SD)	142	73.4 (13.7)	135	72.6 (15.4)	15	75.5 (15.4)	40	76.9 (10.0)
Median (Q1, Q3)		73.3 (63.2, 81.7)		71.3 (61.3, 82.7)		74.0 (67.0, 80.7)		76.8 (70.8, 81.5)
Peripheral vascular disease								
n (%) Yes	145	6 (4.1)	135	5 (3.7)	15	0 (0.0)	40	0 (0.0)
Diabetes								
n (%) Yes	145	59 (40.7)	135	52 (38.5)	15	6 (40.0)	40	15 (37.5)
Hypertension								
n (%) Yes	145	116 (80.0)	135	101 (74.8)	15	11 (73.3)	40	33 (82.5)
Hyperlipidemia								
n (%) Yes	145	39 (26.9)	135	23 (17.0)	15	4 (26.7)	40	5 (12.5)
Previous MI								
n (%) Yes	145	21 (14.5)	135	14 (10.4)	15	0 (0.0)	40	6 (15.0)
Heart failure								
n (%) Yes	145	17 (11.7)	135	14 (10.4)	15	0 (0.0)	40	1 (2.5)
Cerebrovascular events								
n (%) Yes	145	17 (11.7)	135	8 (5.9)	15	1 (6.7)	40	0 (0.0)
Cardiovascular								
n (%) Yes	145	25 (17.2)	135	30 (22.2)	15	2 (13.3)	40	12 (30.0)
Musculoskeletal and orthopedic condition								
n (%) Yes	145	19 (13.1)	135	16 (11.9)	15	1 (6.7)	40	7 (17.5)
Hb								
Mean (SD)	141	110.2 (12.1)	127	109.8 (14.1)	15	118.1 (14.2)	37	108.9 (15.8)
Median (Q1, Q3)		109.0 (103.0, 119.0)		110.0 (102.0, 118.5)		115.0 (109.0, 124.0)		110.0 (100.0, 120.0)
CRP (mg/l)								
Mean (SD)	139	15.3 (21.1)	125	11.9 (15.9)	15	12.5 (16.4)	36	21.1 (26.6)
Median (Q1, Q3)		6.6 (3.1, 18.1)		6.0 (3.0, 14.1)		8.0 (4.5, 11.0)		10.9 (4.3, 28.1)
Dialysis efficiency (%)								
Mean (SD)	141	71.2 (8.4)	125	71.9 (7.3)	15	71.0 (11.3)	37	71.6 (7.9)
Median (Q1, Q3)		72.0 (66.0, 77.0)		73.0 (69.0, 76.5)		74.0 (68.0, 77.8)		71.8 (66.0, 77.0)

BMI, body mass index; CON, control group; CRP, C-reactive protein; DBP, diastolic blood pressure; EX, exercise intervention group; Hb, hemoglobin; MI, myocardial infraction; Q, quartile; SBP, systolic blood pressure.

Continuous variables are revealed as mean (SD) and median (Q1, Q3).

a Indian, Pakistani, and Bangladeshi.

### Effect of Intradialytic Exercise Training on HRQoL

For the primary outcome, the mean difference in the change in PCS from baseline to 6 months between EX and CON was 2.4 (95% CI: −0.1 to 4.8) arbitrary unit and was not statistically significant (*P* = 0.055). Similarly, other measures of HRQoL (energy/fatigue, burden of kidney disease, EQ-5D-5L, and EQ-5D visual analog scale: Table 2; the remaining 7 multi-item scales from the KDQOL-SF: Supplementary Table S1) were all unchanged by the intervention.

**Table 2:** Response of quality of life to the PEDAL intervention, as assessed by KDQOL-SF 1.3 and EQ-5D-5L questionnaires

Outcome measure	*n*^a^	Baseline	Month 6	Adjusted mean difference in change between EX and CON groups^b^	*P* value^c^
Primary outcome					
KDQOL-SF 1.3 PCS (AU)					
CON	120	32.9 (11.3)	31.8 (11.3)	2.4 (−0.1 to 4.8)	0.06
EX	114	33.8 (10.6)	34.8 (11.6)		
Secondary outcomes					
KDQOL-SF 1.3 Energy/fatigue (AU)					
CON	122	39.8 (26.0)	41.4 (24.9)	0.1 (−5.6 to 5.8)	0.97
EX	114	40.3 (27.2)	41.4 (26.4)		
KDQOL-SF 1.3 burden of kidney disease (AU)					
CON	122	36.0 (28.6)	37.3 (29.7)	−1.4 (−7.0 to 4.1)	0.61
EX	113	37.3 (27.7)	36.9 (29.0)		
EQ-5D-5L health utility score (AU)					
CON	121	0.69 (0.25)	0.68 (0.26)	0.01 (−0.04 to 0.07)	0.69
EX	111	0.71 (0.22)	0.70 (0.25)		
EQ-5D visual analog scale (0–100 scale)					
CON	121	59.4 (22.7)	59.3 (20.9)	3.5 (−1.0 to 8.1)	0.13
EX	111	60.7 (22.2)	63.7 (19.3)		

AU, arbitrary unit; CON, control group; EQ-5D, EuroQol 5-dimension descriptive system; EX, exercise intervention group; KDQOL-SF 1.3, Kidney Disease Quality of Life Short-Form; PCS, physical component summary; PEDAL, PrEscription of intraDialytic exercise to improve quAlity of Life.

Data are mean (SD) or mean (95% confidence interval). CON—usual care maintenance hemodialysis. EX—intradialytic exercise training plus usual care maintenance hemodialysis.

a Number of participants with baseline and 6-month data available.

b Adjusting for baseline data and the randomization minimization variables (age, gender, diabetes status).

c Comparison between the control and intervention groups using a normal linear model.

### Cost-Effectiveness

**Figure 2:**
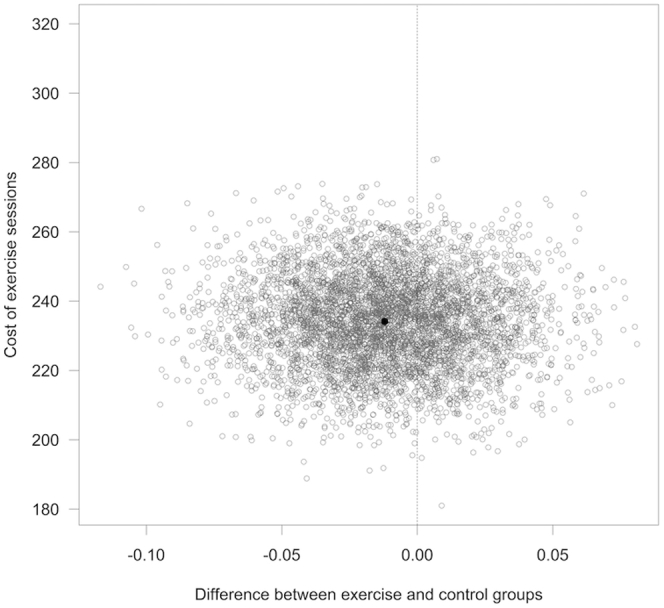
Cost-effectiveness: estimated differences in cost and QALYs on the ICER plane for a low staff-to-patient ratio, outside London (5000 bootstrap samples). ICER, incremental cost effectiveness ratio; QALY, quality-adjusted life year.

The mean (SD) of the area under the EQ-5D-5L curve was 0.665 (0.248) in the CON and 0.653 (0.269) in the intervention group. The mean difference between treatment and intervention groups obtained using the method of recycled predictions was −0.012 (95% CI: −0.069 to 0.043), suggesting no difference in QoL between the intervention and CONs (Figure 2 for an example analysis calculated using a low staff-to-patient ratio, outside London). No significant subgroup effects were found for age, sex, or diabetes at baseline.

Costs from different sources under different scenarios for staff costs are found in Table 3. Average total costs per patient in 6 months range from £232 (US$299) (95% CI: £204–£259) to £424 (US$546) (95% CI: £374–£474), depending on location and staff to patient ratio. The main cost factor was the staff cost for delivering the exercise sessions.

**Table 3:** Costs per patient to deliver the PEDAL intervention in the 6-month follow-up period

Cost source	Outside London		London	
	Low staff-to-patient ratio	High staff-to-patient ratio	Low staff-to-patient ratio	High staff-to-patient ratio
Equipment purchasing and maintenance (£)	9 (8–10)	9 (8–10)	9 (8–10)	9 (8–10)
Staff delivering exercise sessions (£)	204 (180–228)	341 (300–381)	237 (209–265)	395 (348–441)
Training and oversight (£)	18 (16–20)	18 (16–20)	20 (18–23)	20 (18–23)
Total cost per patient in 6 months (£)	232 (204–259)	368 (324–412)	266 (235–298)	424 (374–474)
Estimated difference in cost (recycled predictions) (£)	234 (209–260)	372 (331–414)	269 (240–299)	428 (380–476)

PEDAL, PrEscription of intraDialytic exercise to improve quAlity of Life.

Data are mean (95% confidence interval). Estimated differences in cost obtained by the method of recycled prediction in 5000 bootstrap samples, setting cost in the control group to 0, adjusted for age, sex, and diabetes at baseline.

### Effect of Intradialytic Exercise Training on Secondary Outcomes

Consistent with the lack of change in HRQoL, there were no statistically significant or absolute changes in physical function outcomes (Table 4), cardiovascular risk (arterial stiffness: Table 4), or clinical measures (routine clinical blood tests and medications: data not found). Although mortality was not influenced by the intervention, the number of hospitalizations tended to be higher in the EX group (Table 5). This trend was driven by 11 patients in the EX group who were each hospitalized more than 4 times during the trial for reasons deemed unlikely to be related to the intervention (e.g., fistula issues); in contrast, only 2 patients in the CON group were hospitalized more than 4 times.

**Table 4:** Response of secondary outcome measures to the PEDAL intervention

Outcome measure	*n*^a^	Baseline	Month 6	Adjusted mean difference in change between EX and CON groups^b^	*P* value^c^
Peak aerobic capacity (VO_2_ peak, l/min)					
CON	68	0.97 (0.38)	0.96 (0.37)	0.05 (−0.03 to 0.12)	0.22
EX	75	0.95 (0.42)	0.98 (0.43)		
Peak aerobic capacity (VO_2_ peak, ml/min/kg)					
CON	68	11.9 (4.5)	11.8 (4.2)	0.75 (−0.20 to 1.71)	0.12
EX	74	11.8 (5.3)	12.4 (5.7)		
Arterial stiffness by pulse wave velocity (ms)^22^					
CON	78	8.10 (6.78, 9.29)	7.78 (6.97, 9.13)	1.01 (0.97–1.06)	0.54
EX	78	7.92 (6.62, 9.09)	7.88 (6.98, 9.27)		
DASI (AU)					
CON	121	23.1 (13.1)	22.7 (13.4)	0.35 (−2.23 to 2.93)	0.79
EX	112	24.9 (13.3)	24.1 (14.3)		
IPAQ total physical activity (MET, min/wk) [ln(x + 10)]					
CON	118	423.8 (39.0, 1465.4)	353.2 (46.1, 1033.1)	1.36 (0.84–2.21)	0.21
EX	106	709.5 (153.8, 2515.1)	591.0 (111.8, 1793.2)		
Gait speed in 10 m (m/s)					
CON	84	0.86 (0.30)	0.87 (0.29)	0.01 (−0.04 to 0.06)	0.73
EX	79	0.94 (0.29)	0.94 (0.30)		
Sit-to-stand 60 s (no. of repetitions)					
CON	87	13.8 (6.6)	14.4 (7.0)	1.02 (−0.42 to 2.47)	0.16
EX	82	15.8 (7.1)	17.1 (8.1)		
Tinetti Falls Efficacy Scale (AU) [ln(x)]					
CON	122	22.5 (10.2, 46.8)	24.5 (11.0, 50.0)	0.94 (0.80–1.12)	0.49
EX	112	23.0 (11.8, 49.2)	24.5 (11.0, 46.2)		

AU, arbitrary units; CON, control group; DASI, Duke Activity Status Index; EX, exercise intervention group; IPAQ, International Physical Activity Questionnaire; MET, metabolic equivalent task; PEDAL, PrEscription of intraDialytic exercise to improve quAlity of Life; VO2, maximum rate of oxygen consumption.

Data are mean (SD), median (IQR), or mean (95% confidence interval); some variables were transformed to enhance model fit: transformations are given in [brackets]. CON—usual care maintenance hemodialysis; EX—intradialytic exercise training plus usual care maintenance hemodialysis.

a Number of participants with baseline and 6-month data available.

b Adjusting for baseline data and the randomization minimization variables (age, gender, diabetes status): note variables analyzed as log-transformed values are given as ratios.

c Comparison between the control and intervention groups using a normal linear model.

**Table 5:** Number of hospitalizations and mortality during the PEDAL trial

Variable	*n*^a^	No. of hospitalizations (hospitalization rate per person year)	bIncident rate ratio (95% confidence interval)	*P* value^c^
No. of hospitalizations				
CON	160	84 (0.54)	1.39 (0.93–2.08)	0.109
EX	175	132 (0.85)		

		No. of events (event rate per 100 person years)	dHazard ratio (95% confidence interval)	*P* value
All-cause mortality				
CON	160	9 (5.8)	1.19 (0.48–2.94)	0.71
EX	174	10 (6.5)		
Cardiovascular mortality				
CON	160	3 (1.9)	N/A	N/A
EX	174	2 (1.3)		

CON, control group; EX, exercise intervention group; N/A, not applicable; PEDAL, PrEscription of intraDialytic exercise to improve quAlity of Life.

CON—usual care maintenance hemodialysis. EX—intradialytic exercise training plus usual care maintenance hemodialysis. N/A—as numbers too small to analyze.

a Number of participants with baseline and 6-month data available.

b Incident rate ratios have been calculated in negative binomial regression predicting number of hospitalizations from treatment, adjusting for age, sex, and diabetes at baseline.

c For all-cause mortality, survival was adjusted for age, sex, and diabetes at baseline; for cardiovascular mortality, survival was adjusted for age and diabetes at baseline.

d Hazard ratios have been calculated in Cox proportional hazard regression models predicting survival from treatment.

### Harms

There was no noticeable increase in SAEs in the exercise group (Table 6). Nevertheless, there was 1 noticeable SAE: an individual with type 1 diabetes and autonomic neuropathy experienced severe episodes of symptomatic hypotension that were possibly exacerbated by the intervention. The participant was withdrawn.

**Table 6:** Number of patients with at least 1 SAE by MedDRA system organ class during the PEDAL trial

Variable	All, *n* (%)	CON, *n* (%)	EX, *n* (%)
Number of randomized patients who attended baseline visit	335	160	175
Number of patients with any event	125	56 (35.0)	69 (39.4)
Blood and lymphatic system disorders	2 (0.6)	0 (0.0)	2 (1.1)
Cardiac disorders	15 (4.5)	6 (3.8)	9 (5.1)
Congenital, familial, and genetic disorders	1 (0.3)	1 (0.6)	0 (0.0)
Gastrointestinal disorders	14 (4.2)	4 (2.5)	10 (5.7)
General disorders and administration site conditions	17 (5.1)	12 (7.5)	5 (2.9)
Hepatobiliary disorders	3 (0.9)	1 (0.6)	2 (1.1)
Infections and infestations	47 (14.0)	18 (11.2)	29 (16.6)
Injury, poisoning, and procedural complications	28 (8.4)	12 (7.5)	16 (9.1)
Investigations	5 (1.5)	4 (2.5)	1 (0.6)
Metabolism and nutrition disorders	17 (5.1)	4 (2.5)	13 (7.4)
Musculoskeletal and connective tissue disorders	4 (1.2)	1 (0.6)	3 (1.7)
Neoplasms benign, malignant and unspecified (including cysts and polyps)	1 (0.3)	0 (0.0)	1 (0.6)
Nervous system disorders	8 (2.4)	3 (1.9)	5 (2.9)
Psychiatric disorders	4 (1.2)	1 (0.6)	3 (1.7)
Renal and urinary disorders	1 (0.3)	1 (0.6)	0 (0.0)
Reproductive system and breast disorders	2 (0.6)	1 (0.6)	1 (0.6)
Respiratory, thoracic and mediastinal disorders	13 (3.9)	3 (1.9)	10 (5.7)
Skin and subcutaneous tissue disorders	1 (0.3)	0 (0.0)	1 (0.6)
Social circumstances	1 (0.3)	1 (0.6)	0 (0.0)
Surgical and medical procedures	37 (11.0)	13 (8.1)	24 (13.7)
Vascular disorders	10 (3.0)	6 (3.8)	4 (2.3)

CON, control group; EX, exercise intervention group; MedDRA, Medical Dictionary for Regulatory Activities; PEDAL, PrEscription of intraDialytic exercise to improve quAlity of Life; SAE, serious adverse event.

CON—usual care maintenance hemodialysis. EX—intradialytic exercise training plus usual care maintenance hemodialysis.

### Compliance and Adherence (Fidelity) to the Exercise Prescription

**Figure 3:**
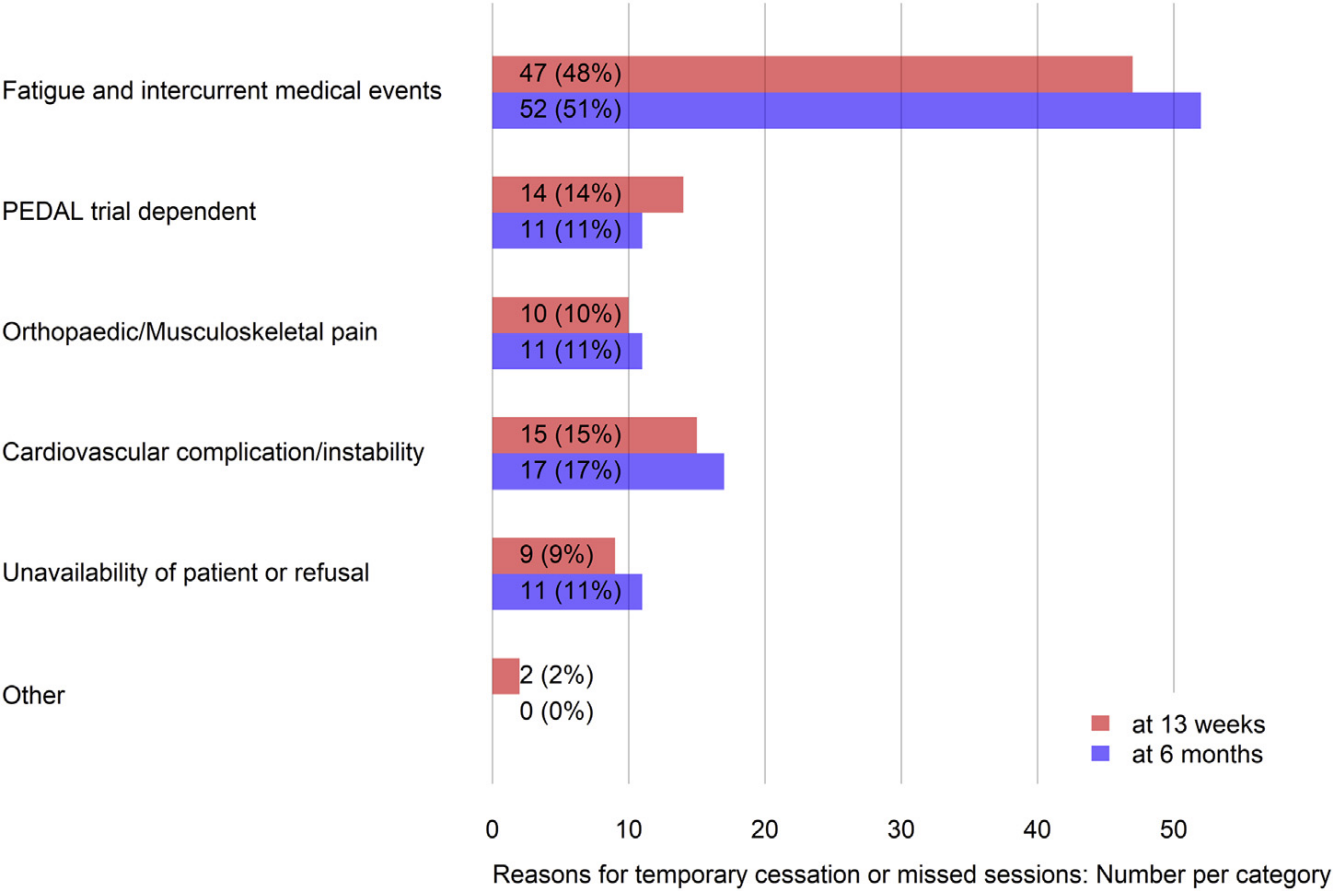
Number (%) of recorded incidents of temporary cessation (>2 weeks) or missed exercise sessions with reasons.

A median (interquartile range) of 47 (28–77)% of exercise training sessions prescribed was completed by participants in EX. Nevertheless, only 18% of patients adhered exactly to the prescribed exercise type, intensity, and duration. Moreover, during the 6-month observation period, only 42% of participants avoided temporary cessation of the exercise intervention (Table 7). Reasons reported were fatigue and intercurrent medical events (Figure 3).

**Table 7:** Summary of exercise compliance and adherence to the PEDAL trial intervention during the 6-month follow-up period

Compliance (percentage of expected sessions completed)	
Sample size (*n*)	175
Median (IQR)	47 (28–77)
Temporary (>2 wk) cessation of exercise	
Sample size (*n*)	119
*n* (%)	69 (58)
Adhered (fidelity to type/intensity/duration) to the exercise prescription	
Sample size (*n*)	119
*n* (%)	21 (18)

IQR, interquartile range; PEDAL, PrEscription of intraDialytic exercise to improve quAlity of Life.

## Discussion

The aim of the PEDAL trial was to evaluate the clinical value of a 6-month intradialytic exercise program on QoL, compared with usual care, for patients receiving HD therapy. The PEDAL trial was novel in that it was the first to evaluate intradialytic exercise as would most likely be implemented, should health service commissioners include exercise training as part of the service specification for in-center HD. Unfortunately, as delivered, the PEDAL program did not statistically improve HRQoL, as assessed by the KDQOL-SF 1.3 PCS (*P* = 0.055), nor did it statistically improve QoL as assessed by the prespecified secondary outcomes of EQ-5D-5L, EQ-5D visual analog scale, or the KDQOL-SF 1.3 multi-item scales of energy/fatigue and burden of kidney disease (Table 2).

The lack of statistical improvement in the PCS can be explained in part by the PEDAL participants having poor compliance (only 47% of prescribed exercise sessions were completed) and very poor adherence (only 18% of patients adhered to the prescribed progression of overload regarding type, intensity, and duration of exercise) to the exercise intervention. By design, the PEDAL trial aimed to have inclusive inclusion criteria. Consequently, baseline peak aerobic capacity values of 12 ml/min/kg were considerably lower than typically reported in previous studies (approximately 18 ml/min/kg).^10^^,^^12^^,^^18^ This observation, combined with extremely low scores in physical performance (sit-to-stand and gait speed tests), confirms that the PEDAL cohort consisted of participants with severely low functional capacity. Arguably, this makes the PEDAL cohort more representative and its findings generalizable to the current HD population. Nevertheless, perhaps including such participants prevented benefits of the exercise intervention being realized in the relatively short 6-month intervention, and it is possible that some of these highly compromised participants may require a slower rate of overload progression and adaptation/adjustment periods to an aerobic intradialytic exercise intervention. Poor compliance and adherence to implemented renal exercise programs in clinical practice is well documented, with more than 50% of the patients starting exercise reportedly dropping out by 6 months, often owing to fatigue and being unwell.^18^^,^^37^^,^^38^

That the PEDAL program was not effective to increase PCS warrants comparison with previous studies. A Cochrane review completed in 2011 concluded that exercise was beneficial for HRQoL in patients with CKD, but unfortunately no meta-analysis or risk of bias assessment was performed, and many of the included studies were not representative of the HD population.^9^ Other reviews have concluded positive effects of exercise but not on PCS^16^^,^^21^^,^^38^ or have relied on studies at high risk of bias and with considerable heterogeneity.^17^^,^^20^ Previous meta-analyses have also included extradialytic exercise programs^19^^,^^39^^,^^40^ and intradialytic exercise programs that were intensively supervised (e.g., Ouzouni *et al.*^41^) or studies that have delivered progressive resistance training as opposed to aerobic cycling alone.^10^^,^^15^^,^^42^ In this regard, 1 meta-analysis^22^ usefully compared aerobic versus progressive resistance training versus combined exercise; only progressive resistance training increased PCS. Detailed analysis of the very few empirical studies included in reviews that do reveal positive effects of aerobic intradialytic exercise on QoL reveals that they have often used interventions that would be difficult to implement in routine care.^41^ A recent study by Jeong *et al.*^43^ found no significant improvements in physical function or QoL with a combined oral protein supplement and intradialytic cycling program. The authors suggested that a more comprehensive lifestyle management approach would be required to elicit improvements in these parameters. Taken together with the results reported herein, it is highly unlikely that clinically implementable intradialytic aerobic exercise training alone can improve QoL at a whole population level.

In addition to evaluating potential benefits, the PEDAL study uniquely assessed the cost of delivery of its intervention by recording harms and using health economic methods. The number of hospitalizations, all-cause mortality, and cardiovascular mortality was not noticeably different between the groups. Although these results should be interpreted cautiously owing to the low number of events, there was no increase in SAE in the exercise group either. The economic cost of delivering the PEDAL intervention ranged from £464 (US$598) to £848 (US$1092) per participant per year (depending on pay band of the physiotherapy assistant, whether London weighting was applied, and staff-to-patient ratio). Note this calculation assumed that physiotherapy assistants supervised between 6 and 10 participants per dialysis session without incurring any travel costs and that exercise would be offered as part of a general physiotherapy service (with enough capacity to provide absence cover at no additional cost). It also assumes that patients will only exercise for between 1 and 2 sessions per week (the calculation is based on compliance to the PEDAL intervention, which was only 47%). For comparison purposes, the cost of delivering cardiac rehabilitation is £477 (US$614) per person per year,^44^ equating to costs of £550 (US$709) to £12,558 (US$16,178) per quality-adjusted life year gained.^45^ In contrast, PEDAL had no apparent QoL gain, albeit in a relatively short period of observation of 6 months (cost-effectiveness of rehabilitation programs increases with time^44^^,^^45^). The cost of delivery of HD in the UK is approximately £35,000 (US$45,088) per patient per year.^46^ As the PEDAL trial was not clinically effective at a whole sample level, whether the cost of delivery of intradialytic exercise is justified to enhance patient choice remains a matter for debate.

### Limitations

PEDAL was designed to assess a pragmatic, clinically implementable intradialytic exercise intervention. By design, the study relied on a patient-reported outcome measure for its primary outcome; it is recognized that the primary limitation of this study was the lack of an attention CON. In this regard, it is possible that an experimenter effect explains the 2.4 arbitrary unit increase (albeit nonsignificant) in PCS.^47^ This interpretation is supported by the lack of absolute or statistical changes in objective measures of physical function, cardiovascular risk, and clinical measures (Table 2), consistent with a conclusion that intradialytic aerobic exercise *per se* had no clinical benefit. In addition, the study was not powered to detect differences in some secondary outcomes, including mortality. Nevertheless, we reported these data to allow a balance of benefits and harms to be evaluated. Future studies should address these concerns by including attention control arms and being adequately powered for all outcome measures. Perhaps the most important finding of the PEDAL study was the observation of poor compliance and adherence when intradialytic exercise was implemented as part of routine care. It is acknowledged that the lack of absolute or significant change in objective measures may in part be due to limitations in the effective implementation of delivering an adequate dose of exercise stimulus as indicated by the very low compliance and adherence data. PEDAL was designed to be a pragmatic intervention, and no additional strategies to address low compliance or adherence were introduced. Thus, future studies need to evaluate whether there are subgroups of patients who may benefit from this type of intervention and whether there is scope to optimize strategies to improve compliance and adherence with intradialytic cycling interventions, implementation settings, and resources to deliver exercise-based interventions to improve effectiveness.

## Conclusions

The PEDAL study was a rehabilitation program that could realistically be commissioned as part of routine care. Compliance and adherence with the exercise intervention, as per the study design, were extremely low. In this inclusive sample of people on HD, many of whom were severely deconditioned; the findings therefore suggest that 6 months of intradialytic aerobic exercise did not improve HRQoL.

## Disclosure

All the authors declared no competing interests.

## Supplementary Material

Supplementary File (PDF)

**Table S1.** Response of quality of life to the PEDAL intervention, as assessed by the Kidney Disease Quality of Life Short-Form (KDQOL-SF 1.3) multi-item scales.

**Consort checklist.**

**STROBE Statement.**
